# Different roles of conserved tyrosine residues of the acylated domains in folding and activity of RTX toxins

**DOI:** 10.1038/s41598-021-99112-3

**Published:** 2021-10-06

**Authors:** Anna Lepesheva, Adriana Osickova, Jana Holubova, David Jurnecka, Sarka Knoblochova, Carlos Espinosa-Vinals, Ladislav Bumba, Karolina Skopova, Radovan Fiser, Radim Osicka, Peter Sebo, Jiri Masin

**Affiliations:** 1 Institute of Microbiology of the Czech Academy of Sciences, Prague, Czech Republic; 2 Department of Genetics and Microbiology, Faculty of Science, Charles University in Prague, Prague, Czech Republic

**Keywords:** Biochemistry, Biophysics, Microbiology

## Abstract

Pore-forming repeats in toxins (RTX) are key virulence factors of many Gram-negative pathogens. We have recently shown that the aromatic side chain of the conserved tyrosine residue 940 within the acylated segment of the RTX adenylate cyclase toxin-hemolysin (CyaA, ACT or AC-Hly) plays a key role in target cell membrane interaction of the toxin. Therefore, we used a truncated CyaA-derived RTX719 construct to analyze the impact of Y940 substitutions on functional folding of the acylated segment of CyaA. Size exclusion chromatography combined with CD spectroscopy revealed that replacement of the aromatic side chain of Y940 by the side chains of alanine or proline residues disrupted the calcium-dependent folding of RTX719 and led to self-aggregation of the otherwise soluble and monomeric protein. Intriguingly, corresponding alanine substitutions of the conserved Y642, Y643 and Y639 residues in the homologous RtxA, HlyA and ApxIA hemolysins from *Kingella kingae*, *Escherichia coli* and *Actinobacillus pleuropneumoniae,* affected the membrane insertion, pore-forming (hemolytic) and cytotoxic capacities of these toxins only marginally. Activities of these toxins were impaired only upon replacement of the conserved tyrosines  by proline residues. It appears, hence, that the critical role of the aromatic side chain of the Y940 residue is highly specific for the functional folding of the acylated domain of CyaA and determines its capacity to penetrate target cell membrane.

## Introduction

The pore-forming RTX leukotoxins and hemolysins of Gram-negative pathogens penetrate and permeabilize host cell membranes^1,2^. Among the best studied RTX leukotoxins is the RTX adenylate cyclase toxin-hemolysin (ACT, AC-Hly or CyaA) of *Bordetella* that plays an important role in the airway colonization capacity of the whooping cough agent *B. pertussis*^3,4^. CyaA comprises an N-terminal cell-invasive adenylyl cyclase enzyme domain (AC, ~ 384 residues) that is linked to a ~ 1322 residue-long C-terminal RTX hemolysin (Hly) moiety^5^. The Hly consists of several functional subdomains. It bears (i) a unique N-terminal ‘AC-to-Hly-linking segment’ (LS, residues ~ 400–500) that participates in membrane translocation of the AC domain of CyaA^6–9^. This is (ii) followed by a hydrophobic pore-forming domain (PFD) of the Hly (residues 500–700) that consists of five hydrophobic and amphipathic transmembrane α-helices^10–15^. The PFD is followed (iii) by an acylated segment (AS, residues 700–1000), where the protoxin is activated through posttranslational covalent palmitoylation of the ε-amino groups of the internal lysine residues K860 and K983, contained within conserved RTX acylation sites and recognized by the dedicated CyaC acyltransferase^16–18^. Finally, (iv) the C-terminal potion of Hly comprises the calcium-binding nonapeptide repeat domain (RTX) that harbors ~ 40 calcium-binding sites and upon Ca^2+^ loading folds into five β-roll blocks^19,20^.

Except for the presence of the unique ‘AC-to-Hly-linking segment’, the domain structure of the Hly moiety of CyaA exemplifies the general domain organization of other known RTX hemolysins/cytolysins that share a fair extent of sequence homology and differ primarily in the number of repeat sequences forming their RTX domains^1^.

The Hly moiety of CyaA binds the complement receptor 3 (CR3, α_M_β_2_ integrin CD11b/CD18 or Mac1) of myeloid phagocytes^21–24^, penetrates the plasma membrane of cells and translocates into their cytosol the N-terminal adenylyl cyclase (AC) enzyme domain^25^. As a result, the AC domain translocates directly from the lipid rafts of the plasma membrane into cytosol of cells without the need for endocytosis^26^. Recently, it was proposed that AC domain translocation is facilitated by interaction of the membrane-penetrating ‘AC-to-Hly linking segment’ with cytosolic calmodulin^9^. Inside cells, the AC enzyme itself binds calmodulin with high affinity and catalyzes uncontrolled conversion of cytosolic ATP into the second messenger molecule cAMP^27^. In parallel, membrane penetration of Hly permeabilizes the plasma membrane of cells, forming small cation-selective (hemolytic) pores that enable potassium efflux from cells and can provoke colloid-osmotic (oncotic) cell lysis at increased CyaA concentrations^28–30^.

Another well studied prototypic RTX toxin is the α-hemolysin (HlyA) secreted by a number of pathogenic as well as commensal isolates of *Escherichia coli*. HlyA forms transmembrane pores in eukaryotic membranes that can provoke oncotic cell lysis, whereas at sublytic concentrations the membrane permeabilizing activity of HlyA hijacks host cell signaling pathways and can trigger apoptotic cell death^31^. Two protein receptors of HlyA have been identified, the α_L_β_2_ integrin LFA-1 (also known as CD11a/CD18) of leukocytes^32,33^ and a glycophorin found in the membrane of human and equine erythrocytes^34^. Yet another homologous pore-forming hemolysin, RtxA, is secreted by the emerging pathogen *Kingella kingae*^35^ and has been implicated in the development of osteoarticular infections and infective endocarditis in children and adults^36^. The hemolysin ApxIA is then produced by *Actinobacillus pleuropneumoniae*, the etiological agent of porcine pleuropneumonia^1^ In contrast to CyaA, which forms small transmembrane pores of only 0.6–0.8 nm in diameter^37^, the HlyA, RtxA and ApxIA hemolysins generate larger pores with an inner diameter of ~ 2 nm^2,38^.

The two prototypical RTX toxins-hemolysins, HlyA and CyaA, preferentially bind leukocytes through the β_2_-integrin receptors^23,32,39^. The initial binding interaction depends on rather unspecific recognition of N-linked oligosaccharides of the integrins^40,41^. Both toxins can, hence, promiscuously bind with low affinity also glycans of other cell surface glycoproteins and glycolipids. As a result, CyaA and Hly can penetrate with a reduced efficacy also various non-leukocytic cells lacking the β_2_-integrins. The cell penetration is a two-step process, where the reversible initial electrostatic adsorption to cells surface glycans is followed by an irreversible membrane insertion of the toxin^42–44^. However, the role of the individual domains of RTX toxins in membrane penetration is poorly understood. Several studies showed that the hydrophobic, acylated and calcium-binding domains synergize in the process of insertion of RTX toxins into the lipid bilayer of target cell plasma membrane^11,45–47^. Deletion of individual segments of the hemolysin portion largely blocks insertion of CyaA into erythrocyte membrane, suggesting that the structural integrity and correct positioning of the individual domains on  the lipid bilayer is essential for membrane insertion of CyaA^48,49^. The integrity of the hydrophobic domains of RTX cytolysins then appears to be crucial for their ability to oligomerize into cation-selective transmembrane pores^10,12,13,15,50–52^ that exhibit a short lifetime of only few seconds^28^.

Recently, we found that the aromatic ring of the side chain of a conserved tyrosine residue 940 (Y940) in the acylated domain of CyaA plays a key role in the membrane penetration capacity of the toxin^12^. Here, we investigated the role of this conserved tyrosine residue in the folding and action of several prototypical RTX hemolysins and show that it is specifically crucial for folding and activity of CyaA.

## Results

### The aromatic ring of the tyrosine residue 940 plays a crucial role in the biological activity of *Bordetella* CyaA toxin

**Figure 1:**
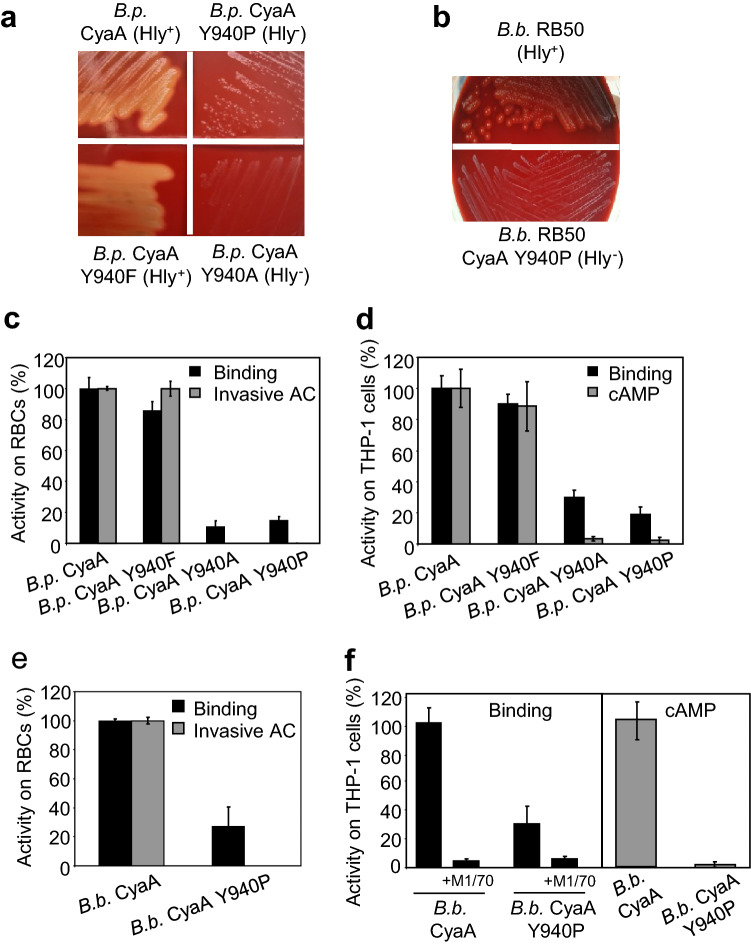
The activity of *B. pertussis (B.p.)* and *B. bronchiseptica (B.b.)* CyaA depends on the structural integrity of the acylated segment. (**a**) Parental *B. pertussis* strain, the fully-hemolytic *B. pertussis* expressing the CyaA Y904F, and the non-hemolytic *B. pertussis* expressing the CyaA Y940A or CyaA Y940P were grown on BG agar with 15% defibrinated sheep blood for 5 days at 37 °C. (**b**) The parental *B. bronchiseptica* RB50 and the non-hemolytic *B. bronchiseptica* expressing CyaA Y940P were grown on BG agar with 15% defibrinated sheep blood for 2 days at 37 °C. (**c**) Sheep erythrocytes were incubated at 37 °C with urea extracts (20 mU of *B. pertussis* CyaA/ml). After 30 min, aliquots were taken to determine the cell-binding and invasive AC. (**d**) The binding of CyaAs to THP-1 cells was determined as the amount of cell-associated AC enzyme activity upon incubation of cells with 40 mU/ml of the protein for 30 min at 4 °C. The cAMP concentration was measured in THP-1 cells (10^5^) after 30 min of incubation with diluted extracts containing 20, 10, 5 and 2.5 mU/ml of CyaA. (**e**) Sheep erythrocytes were incubated at 37 °C with extracts (20 mU of *B. bronchiseptica* CyaA/ml) and activities were analyzed as above. (**f**) Binding of intact *B. bronchiseptica* CyaA or its mutant variants to THP-1 cells was determined as above with 20 mU/ml of the protein. To block the CD11b/CD18, THP-1 cells were pre-incubated for 15 min on ice with 5 μg/ml of the CD11b-specific monoclonal antibody M1/70 prior to the addition of the *B. bronchiseptica* CyaA variants. The intracellular cAMP was measured in THP-1 cells (1.5 × 10^5^) after incubation with diluted bacterial lysates containing 10, 5, 2.5 and 1.25 mU/ml of CyaA. Activities in panels C, D, E and F are expressed as percentages of intact *B. pertussis* or *B. bronchiseptica* CyaA activity and represent mean ± SD of three independent determinations performed in duplicate.

We have previously observed that alanine or proline substitutions of the tyrosine residue 940 (Y940) in the acylated domain of CyaA did not interfere with the CyaC-mediated posttranslational acylation of the toxin, but still ablated the capacity of recombinant CyaA toxin to penetrate target cell membrane and deliver its AC domain into cells^12^. It was thus important to exclude that this loss of toxin activity might be an artifact caused by extraction of the recombinant CyaA proteins from *E. coli*-produced inclusion bodies by denaturating solutions of 8 M urea and subsequent calcium-driven refolding of the recombinant toxin variants in target cells suspensions. Therefore, we examined whether the Y940 substitutions will affect also the activities of CyaA toxin variants secreted by *Bordetella pertussis* and *B. bronchiseptica*. Towards this aim, mutations encoding the various Y940 substitutions were introduced by allelic exchange into the *cyaA* genes on *B. pertussis* and *B. bronchiseptica* chromosomes and the corresponding variants of secreted CyaA toxins were extracted from bacterial cell surface and used in toxin activity assays. Both *B. pertussis* and *B. bronchiseptica* intact CyaAs showed a similar capacity to deliver the AC domain across the plasma membrane of sheep erythrocytes and human THP-1 monocytes (Supplementary Figure S1). As further shown in Fig. 1a,b, the wild-type bacteria producing intact CyaA, or the mutant secreting the CyaA-Y940F toxin variant, exhibited a hemolytic phenotype on Bordet-Gengou (BG) agar plates. In contrast, the *B. pertussis* and *B. bronchiseptica* mutants secreting the CyaA-Y940A or CyaA-Y940P toxins formed non-hemolytic colonies indicative of loss of hemolytic activity the Y940A and Y940P CyaAs. Indeed, when the respective secreted toxins were extracted from the surface of producing bacteria, only the CyaA and the CyaA-Y940F toxin variant were able to bind and penetrate erythrocytes or CR3-expressing THP-1 macrophages in the in vitro activity assay and the CyaA-Y940A and CyaA-Y940P proteins were inactive (Fig. 1c–f). Finally, the loss of biological activity of CyaA upon Y940A and Y940P substitutions was examined by in vivo intranasal infection experiments in Balb/cByJ mice. As shown in Table 1, the mutant *B. pertussis* strains secreting the CyaA Y940A and CyaA Y940P toxin variants exhibited a decreased virulence with about an order of magnitude higher LD_50_ value (lethal dose for 50% of infected animals) than CyaA or CyaA-Y940F -secreting *B. pertussis* strains. All these results thus demonstrate that the presence of the aromatic ring as the side chain of residue 940 of the CyaA protein is critical for the cytotoxic activity of CyaA on target cells in vitro and for its biological function as a key virulence factor of *B. pertussis *in vivo in the mouse model of lung infection.

**Table 1:** The aromatic ring at position 940 is required for the biological activity of CyaA secreted by *B. pertussis* during in vivo respiratory infection of Balb/cByJ mice.

strain^a^	LD_50_^b^
*B.p.* CyaA	1.7 × 10^7^
*B.p.* CyaA Y940F	3.8 × 10^7^
*B.p.* CyaA Y940A	1.8 × 10^8^
*B.p.* CyaA Y940P	5.8 × 10^8^

^a^*B. pertussis* strains were grown in SSM as described in Methods.

^b^Balb/cByJ mice were intranasally infected with individual *B. pertussis* strains to determine LD_50_ values (CFU/mouse) as described in detail in Methods.

### The aromatic side chain of the tyrosine residue 940 is essential for folding of the acylated domain of CyaA

**Figure 2:**
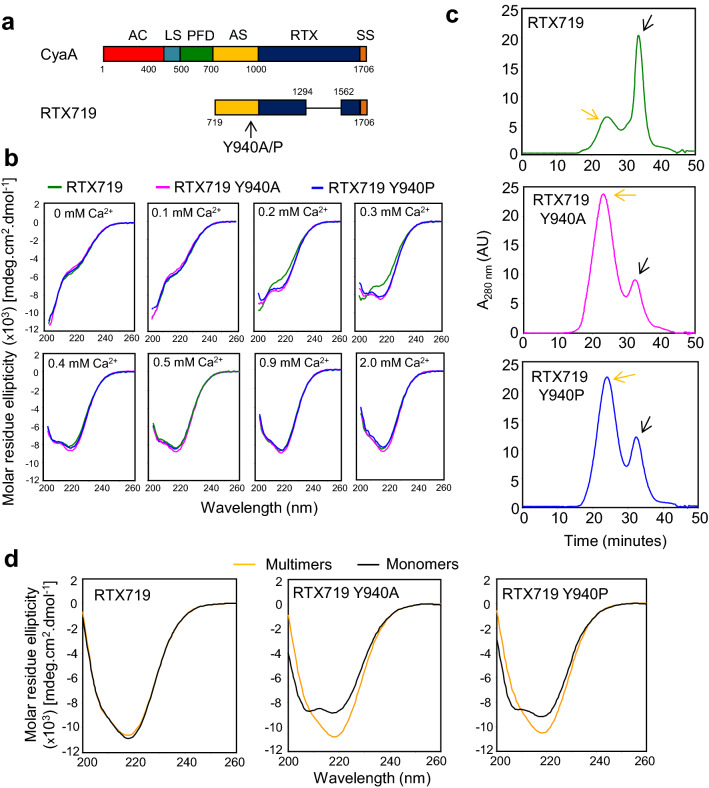
The Y940 residue plays a key structural role in the folding of the acylated segment of CyaA (**a**) Schematic representation of the CyaA and CyaA-derived RTX719. AC, adenylate cyclase domain; LS, AC-to-Hly linker segment; PFD, pore-forming domain; AS, acylated segment; RTX, calcium-binding repeats; SS, secretion signal. (**b**) Far-UV CD spectra of the RTX719, RTX719 Y940A and RTX719 Y940P proteins (200 μg/ml) in the absence and the presence of CaCl_2_. The data correspond to the average of two independent experiments with 3 accumulations per spectrum. (**c**) Gel filtration chromatography was performed on a Superdex 200HR gel filtration column. The column was equilibrated with a buffer containing 20 mM Tris–HCl (pH 8.0), 150 mM NaCl and 2 mM CaCl_2_ before the denatured proteins (400 µg in 50 mM Tris–HCl pH 8.0, 8 M urea and 300 mM NaCl) were loaded onto the column and refolded at a flow rate of 0.5 ml/min. The orange and black arrow indicates multimeric and monomeric state of CyaA constructs, respectively. (**d**) Far-UV CD spectra of the multimeric and monomeric forms of the RTX719, RTX719 Y940A and RTX719 Y940P proteins (100 µg/ml) in 20 mM Tris–HCl (pH 8.0), 150 mM NaCl and 2 mM CaCl_2_.

We hypothesized that substitutions of Y940 by alanine or proline residues affects the folding and formation of a functionally important structure in the CyaA molecule. To test this hypothesis and analyze the structural consequences of Y940 substitution, we constructed a truncated CyaA-derived polypeptide RTX719 (Fig. 2a), in which the fatty acyl-modified segment of CyaA comprising the Y940 residue was fused to a truncated RTX domain capable of vectorial calcium-driven folding^22^. Subsequently, the Y940A and Y940P substitutions were introduced into the RTX719 construct, the proteins were produced in *E. coli* cells in the presence of the CyaC acyltransferase and purified close to homogeneity from urea extracts by chromatography on DEAE-Sepharose (Supplementary Figure S2). The calcium-dependent folding of the RTX719 variants was then analyzed by circular dichroism (CD) spectroscopy. The urea-denatured proteins were refolded in the Ca^2+^-free buffer and titrated by stepwise addition of Ca^2+^ ions. The far-UV CD spectra revealed that the RTX719 construct as well as its Y940A and Y940P variants undergo a Ca^2+^-dependent structural transition from an unfolded to a folded conformation that is characterized by a prominent negative peak in the spectra at 218 nm, which corresponds to a parallel β-roll structure (Fig. 2b). Intriguingly, the Ca^2+^-induced assembly of the Y940A and Y940P mutants was initiated and reached completion at lower Ca^2+^ ion concentrations, as that needed for completion of folding of the intact RTX719 construct (Fig. 2b, Supplementary Figure S3). Moreover, the on-column refolding characteristics of the RTX719 variants during Superdex HR200 gel permeation chromatography differed substantially. As documented in Fig. 2c, a major fraction of the on-column refolded RTX719 eluted as monomeric protein with a retention time of 32 min, while the Y940A and Y940P proteins formed predominantly oligomers eluting with retention time of 25 min. Moreover, the far-UV CD spectrum of the eluted RTX719 monomers exhibited a single negative peak at 218 nm typical of the β-stranded protein skeletons (Fig. 2d). In contrast, the spectra of the monomeric forms of the Y940A and Y940P protein variants eluted in the minor fraction at 32 min (black arrows over chromatograms in Fig. 2c and black curves in Fig. 2d) exhibited two negative peaks in the spectra at 206 and 218 nm, revealing the presence of a mixture of α-helices and β-sheets in their structures. Taken together, these data clearly indicated that the conserved tyrosine 940 residue plays a key role in Ca^2+^-induced folding of the acylated domain of CyaA and its replacement by a non-aromatic residue results in misfolding and aggregation of the RTX719 construct.

### Substitutions of the conserved tyrosine residues in the acylated segments of other RTX hemolysins do not affect their posttranslational acylation but proline substitutions impair their toxin activites

**Figure 3:**
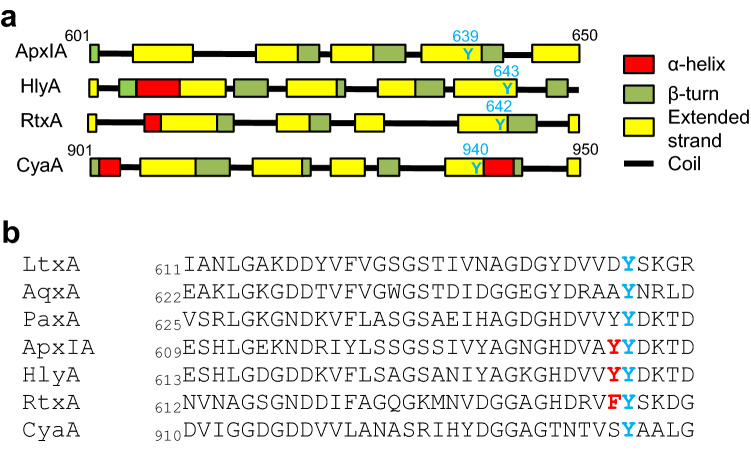
A conserved tyrosine residue is present in the acylated segment of RTX toxins. (**a**) Prediction of secondary structures in CyaA (residues 901–950) and 601–650 of ApxIA, HlyA and RtxA was performed using SOPMA software^71^. Highly conserved Y639 from ApxIA, Y643 from HlyA, Y642 from RtxA and Y940 from CyaA are highlighted by a blue color. (**b**) ClustalW sequence alignment of a partial sequence of the acylated segment of CyaA and corresponding sequences of related RTX toxins. LtxA, *Aggregatibacter actinomycetemcomitans* (UniProtKB P16462); AqxA, *Actinobacillus equuli* (UniProtKB Q8KWZ9); PaxA, *Pasteurella aerogenes* (UniProtKB Q9RCG8); ApxIA, *Actinobacillus pleuropneumoniae* (UniProtKB P55128); HlyA, *Escherichia coli* (UniProtKB P08715); RtxA, *Kingella kingae* (UniProtKB A0A1X7QMH9); and CyaA, *Bordetella pertussis* (UniProtKB P0DKX7). The highly conserved residues Y639 of ApxIA, Y643 of HlyA, Y642 of RtxA and Y940 of CyaA are highlighted by a blue color. The non-conserved Y638 of ApxIA, Y642 of HlyA and F641 of RtxA are highlighted by a red color.

The Y940 residue is located in a predicted β-strand structure within the acylated segment of CyaA and appears to be highly conserved within the homologous segments of other pore-forming RTX hemolysins (Fig. 3). To analyze whether the conserved tyrosine residues might play a role also in the functional folding and cytotoxic activities of other hemolysins, we replaced the Y642 residue of RtxA from *Kingella kingae*, the Y643 residue of HlyA from *Escherichia coli*, and the Y639 residue of ApxIA from *Actinobacillus pleuropneumoniae* with phenylalanine, alanine, and proline residues, respectively. The toxin variants were produced in *E. coli* BL-21 cells and purified close to homogeneity from urea extracts by Ni–NTA chromatography using the purpose-introduced 6xHis affinity purification tags. As verified by tandem mass spectrometry (Supplementary Table S1), the substitutions of the conserved tyrosine residues did not affect the ability of the co-expressed cognate acyltransferase RtxC, HlyC, and ApxIC to recognize and posttranslationally acylate the respective segments of proRtxA, proHlyA, and proApxIA, which were quantitatively modified by myristoylation and hydroxymyristoylation on the corresponding lysine residues.

**Figure 4:**
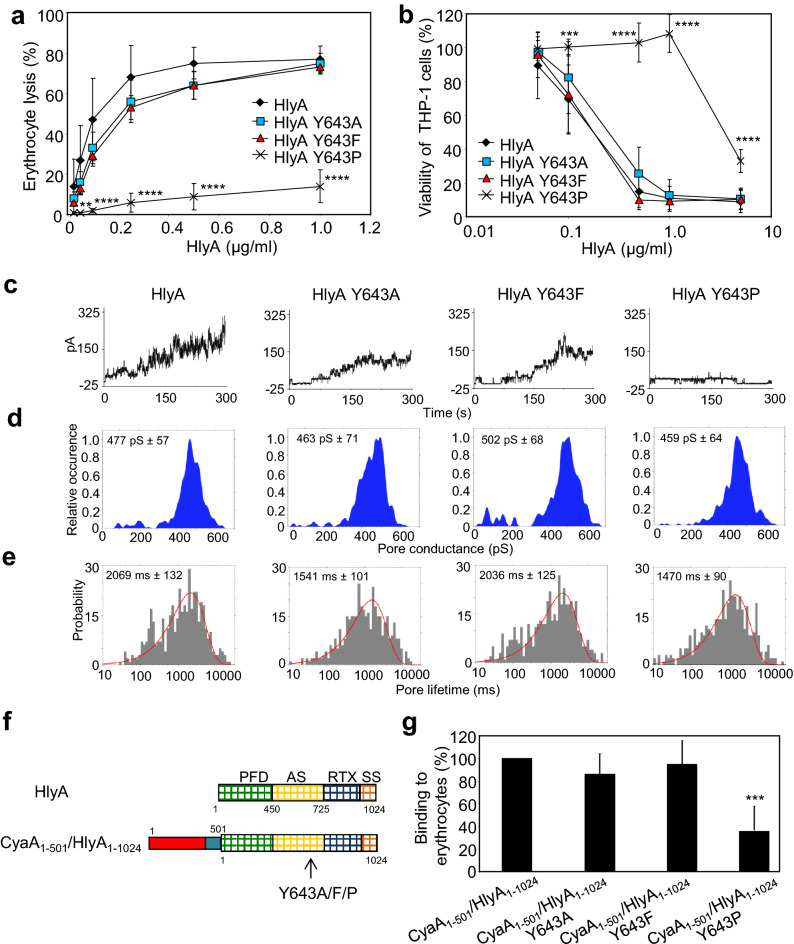
The Y643P substitution blocks membrane insertion of HlyA. (**a**) Erythrocytes were incubated at 37 °C in TNC buffer in the presence of various concentrations of purified HlyA. Hemolytic activity was measured after 20 min as the amount of hemoglobin released (A_541nm_). Complete lysis of erythrocytes was expressed as 100%. Each point represents the mean ± SD of six independent determinations performed in triplicate with three independent toxin preparations. (**b**) Cell viability of HlyA-treated THP-1 cells (1.5 × 10^5^/well) was determined as the capacity of mitochondrial dehydrogenases to reduce the tetrazolium salt WST-1 to its formazan product after 2 h at 37 °C. Each point represents the mean ± SD of at three independent determinations performed in triplicate with two independent toxin preparations. (**c**) Overall membrane activities of the HlyA variants on asolectin/decane:butanol (9:1) membranes in the presence of 3 nM purified proteins. The aqueous phase contained 150 mM KCl, 10 mM Tris–HCl (pH 7.4), 2 mM CaCl_2_; the applied voltage was 50 mV; the temperature was 25 °C; and the recording was filtered at 100 Hz. (**d**) Kernel density estimation (KDE) of single-pore conductances calculated from single-pore recordings (> 1,000 events) acquired on several different asolectin membranes with 3 nM HlyA variants under the same conditions as in C. (**e**) For lifetime determination, 450–460 individual pore openings were recorded on several different asolectin membranes with 1 and 3 nM concentrations of HlyA variants under the same conditions as in C. (**f**) Schematic representation of the intact HlyA and the CyaA_1-501_HlyA_1-1024_ hybrid protein, containing the AC domain and linker segment (residues 1–501, Fig. 2a) fused to intact HlyA (residues 1–1024) or HlyA bearing the substitution of the conserved Y643 by a proline, alanine, or phenylalanine residue. Individual domains of CyaA and HlyA are indicated by the colored rectangles. PFD, pore-forming domain; AS, acylated segment; RTX, calcium-binding repeats; SS, secretion signal. (**g**) Sheep erythrocytes (5 × 10^8^/ml) were incubated in TNC buffer in the presence of 75 mM sucrose as osmoprotectant at 37 °C with 0.4 U/ml of urea extract of the hybrid proteins and after 5 min, aliquots were taken for determinations of the cell-associated AC activity. The binding capacity of HlyC-activated CyaA_1-501_HlyA_1-1024_ was expressed as a percentage of intact HlyC-activated CyaA_1-501_HlyA_1-1024_ activity and represents mean ± standard deviations of four independent determinations with two different toxin preparations. Significant differences are indicated by asterisks (**p < 0.01; ***p < 0.001; ****p < 0.0001).

As shown in Fig. 4a, the HlyA Y643A and HlyA Y643F variants exhibited a near intact hemolytic activity on sheep erythrocytes, whereas the activity of the HlyA Y643P construct was strongly reduced over a wide range of toxin concentrations. Similarly, the cytotoxicity of the HlyA Y643P variant towards LFA-1-positive THP-1 cells was markedly reduced and its cytotoxic effect was observed only at the highest concentration used, whereas the cytotoxic capacity of the HlyA Y643A and HlyA Y643F variants was similar to that of intact HlyA toxin (Fig. 4b). Consistent with this, the HlyA, HlyA Y643A, and HlyA Y643F proteins exhibited similar overall membrane activity on artificial lipid bilayers prepared from crude asolectin, whereas the HlyA Y643P variant formed pores with a significantly lower overall membrane activity than intact HlyA (Fig. 4c). As shown in Fig. 4d,e, the pore-characteristics, such as mean single pore conductance and lifetime of pores formed by all three HlyA mutants were comparable to those of intact HlyA. These data indicated that the presence of the aromatic ring in the side chain of the conserved Y643 residue of HlyA was as such not essential for the formation of the α-hemolysin pores, since the HlyA Y643A variant and the intact HlyA toxin exhibited comparable pore-forming membrane activities.

We further analyzed whether the low pore-forming (hemolytic) and cytotoxic potency of the HlyA Y643P variant was due to a reduced membrane-binding capacity. Towards this aim, we prepared a series of HlyC-activated chimeric molecules in which the full-length HlyA (residues 1–1024) carrying the substitutions Y643A, Y643F, or Y643P was fused to the AC domain and the AC-to-Hly linker segment of CyaA (N-terminal residues 1–501), (Fig. 4f). The highly active N-terminal AC enzyme domain in the hybrid proteins then allowed the quantification of the specific capacity of the molecules to associate tightly with the cell membrane. As shown in Fig. 4g, the cell binding capacity of CyaA_1-501_/HlyA_1-1024_ with the Y643F and Y643A substitutions was almost indistinguishable from the cell binding capacity of the intact chimera. In contrast, the cell binding capacity of the CyaA_1-501_/HlyA_1-1024_ Y643P construct was reduced by ~ 70% compared to the intact chimera. These data strongly suggest that the low pore-forming and cytolytic activity of the HlyA Y643P mutant was due to the reduced membrane association capacity of the construct.

### A tyrosine residue pair plays a role in membrane penetration of HlyA

In contrast to CyaA, where the conserved Y940 is adjacent to a hydrophilic S939 residue, a pair of hydrophobic aromatic residues is present in the acylated segment of HlyA (Fig. 3b). To analyze whether the adjacent Y642 residue synergizes with the conserved Y643 residue in maximizing the lytic activity of HlyA on target cells, we produced HlyA-derived constructs carrying both tyrosine residues substituted by a pair of alanine (HlyA Y642A + Y643A) or proline (HlyA Y642P + Y643P) residues, and a further construct had the Y642 replaced by a proline residue (HlyA Y642P).

**Figure 5:**
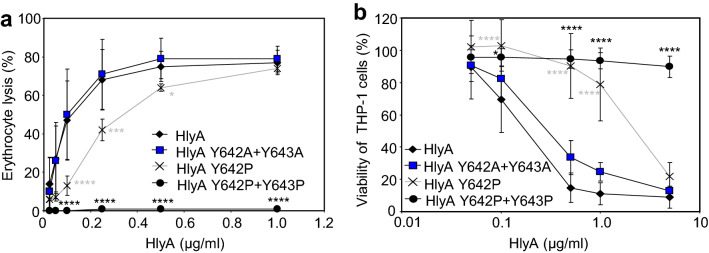
A tyrosine residue pair plays a role in cytotoxic activity of HlyA. Hemolytic activity of HlyA variants (**a**) was analyzed as detailed in Fig. 4. Each point represents the mean ± SD of three independent determinations performed in duplicate with two different toxin preparations. (**b**) Cell viability was analyzed on THP-1 cells as detailed in Fig. 4. Each point represents the mean ± SD of three independent determinations performed in triplicate with two different toxin preparations. Significant differences are indicated by asterisks (*p < 0.05; ***p < 0.001; ****p < 0.0001).

As shown in Fig. 5, the hemolytic and cytotoxic activities of intact HlyA and of the doubly substituted HlyA Y642A + Y643A variant were similar, suggesting that there was no functional impact of a combined tyrosine-to-alanine substitution at the positions 642 and 643 of HlyA. However, the hemolytic and cytotoxic activities of the HlyA Y642P protein were reduced over a range of toxin concentrations (Fig. 5). Moreover, the Y642P + Y643P double substitution completely abolished the cytolytic activity of the fully acylated HlyA Y642P + Y643P protein (Supplementary Table S1) on both erythrocytes and THP-1 macrophages (Fig. 5). In conclusion, the nil effect of the double Y642A + Y643A substitutions on HlyA cytotoxic activities strongly suggests that the aromatic side chains of Y642 and Y643 are not critical for membrane insertion and subsequent lytic activity of HlyA. Intriguingly, the complete loss of toxin activity occurred only upon a double Y642P + Y643P substitution, presumably destroying the local structure of the HlyA segment.

### A single substitution of the tyrosines does not affect the formation of secondary structures of the acylated segment of HlyA

**Figure 6:**
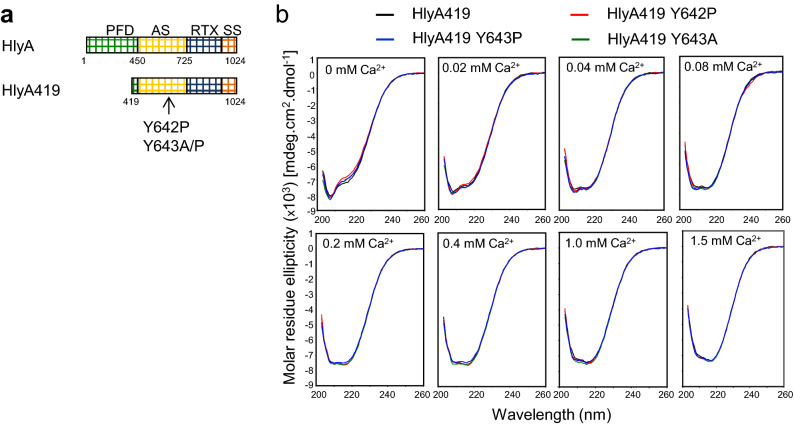
Substitution of the tyrosine of the acylated segment did not affect the formation of secondary structures of the HlyA419 (**a**) Schematic representation of the HlyA and HlyA-derived HlyA419. PFD, pore-forming domain; AS, acylated segment; RTX, calcium-binding repeats; SS, secretion signal. (**b**) Far-UV CD spectra of the HlyA419, HlyA419 Y642P, HlyA419 Y643P and HlyA419 Y643A proteins (200 μg/ml) in the absence and the presence of CaCl_2_. The data correspond to the average of two independent experiments with 3 accumulations per spectrum.

To assess the structural impact of tyrosine residue substitutions in the acylated domain of HlyA, we constructed a truncated HlyA-derived polypeptide carrying the C-terminal residues 419–1024 (HlyA419, Fig. 6a). Subsequently, the Y642P, Y643P and Y643A substitutions were introduced into the HlyA419 construct and the corresponding proteins were produced in *E. coli* in the presence of HlyC acyltransferase and purified from the urea extracts on Ni–NTA Sepharose (Supplementary Figure S4). Far-UV CD spectra of the Hly-derived constructs revealed that all four proteins undergo a similar Ca^2+^-dependent structural change from disordered conformation to β-roll secondary structures (Fig. 6b). These data show that in contrast to a similar CyaA719 construct, the substitutions of the tyrosine residue of the acylated segment of HlyA with alanine or proline residue do not affect any importantly the Ca^2+^-dependent formation of secondary structures in the truncated HlyA419 fragment.

### Proline, but not alanine substitutions of the conserved tyrosine residues strongly impair the hemolytic activity of the RtxA and ApxIA hemolysins

**Figure 7:**
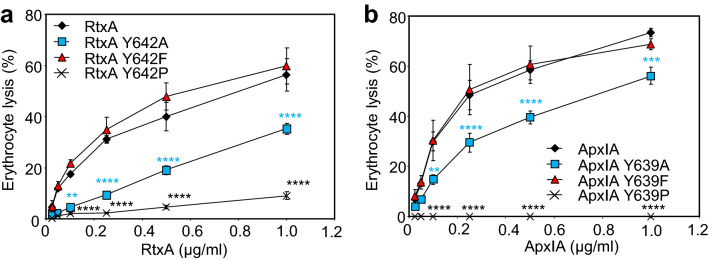
The aromatic ring of the conserved tyrosine is essential for the full hemolytic activity of RtxA and ApxIA. The hemolytic activity of RtxA (**a**) and ApxIA (**b**) was analyzed as described in Fig. 4. Each point represents the mean ± SD of three independent determinations performed in duplicate with two independent toxin preparations. Significant differences are indicated by asterisks (**p < 0.01; ***p < 0.001; ****p < 0.0001).

Finally, the hemolytic activity of the RtxA and ApxIA hemolysins bearing susbtitutions of the conserved tyrosine residues of the acylated domains were analyzed. As shown in Fig. 7a, while the Y642F variant of RtxA was fully active and the Y642A substitution affected the hemolytic activity of RtxA only modestly, the capacity of the RtxA Y642P construct to lyse erythrocytes was strongly impaired. Similarly, the ApxIA Y639P construct was unable to lyse erythrocytes even at high toxin concentrations, while the hemolytic activity of the ApxIA Y639A variant was reduced by a factor of ~ 2 compared to the intact toxin or its ApxIA Y639F variant (Fig. 7b).

In summary, these data indicate that the aromatic side chain of the conserved Y642 and Y639 residue is important for the full hemolytic activity of RtxA and of ApxIA hemolysins on sheep erythrocytes.

## Discussion

We show here that the aromatic ring of the side chains of the conserved tyrosine residues located in the acylated segments of four RTX toxins play a different roles in their biological activities. While replacement of the aromatic ring of Y940 residue of CyaA by the alanine or proline residues led to self-aggregation of the truncated RTX719 protein and loss of toxin activities of the full-length CyaA, the elimination of the tyrosine residue at position 643 of HlyA (Y643A) did not significantly affect the folding of the acylated segment and subsequent cytotoxic activity of the α-hemolysin. Interestingly, for the quite homologous RtxA and ApxIA hemolysins, the presence of the aromatic ring of the conserved tyrosine residues were still required for a full hemolytic activity of the RtxA (Y642A) and of ApxIA (Y639A) toxins.

First, we re-examined the toxin activities of the CyaA variants secreted by corresponding mutant strains of the closely related *B. pertussis* and *B. bronchiseptica* This was important to do, as CyaA is translocated from the cytosol of *Bordetella* directly into the external medium through a T1SS channel-tunnel assembly spanning the bacterial envelope and undergoes a vectorial co-secretional folding initiated by the first-emerging C-terminal folding scaffold upon exit from the bacterial cell due to binding of extracellular calcium ions^19^. It was thus crucial to verify that also such excreted and vectorially folded molecules of CyaA bearing the Y940A and Y940P substitutions will be devoid of membrane penetrating activity, like the recombinant forms of the toxin produced and acylated in *E. coli* cells. Indeed, as shown in Fig. 1, this was the case for the corresponding mutant forms of CyaA secreted by both *Bordetella* species.

Recently, Fukui-Miyazaki and coworkers presented that *B. bronchiseptica* CyaA, in contrast to *B. pertussis* CyaA, exhibits weak cAMP intoxication of nucleated cells due to phosphorylation of the S375 residue that is bound by the host factor 14-3-3 and results in abrogation of the AC penetration activity^53^. Working with the same human THP-1 cell line and parental *B. bronchiseptica* RB50 strain as Fukui-Miyazaki et al., we were unable to reproduce the reported striking difference in the specific capacity of *B. pertussis* and *B. bronchiseptica* CyaA proteins to increase cAMP in target cell cytosol and we found that the two proteins exhibited comparable cAMP-elevating activity in macrophage cells, as previously also reported by Henderson and colleagues^54^. Moreover, using LC FT-ICR-MS analysis, we checked the amino acid residues at position 375 of purified CyaAs by peptide mass mapping (Supplementary Figure S5) and found that *B. pertussis* CyaA carried F375, whereas *B. bronchiseptica* CyaA carried S375, as previously described^53^. Our mass spectrometry analysis also identified other substitutions in the CyaA sequences, namely at positions 370, 800, 808, 910, and 978, located in the N-terminal part of *B. pertussis* and *B. bronchiseptica* CyaAs (Supplementary Figure S5), confirming that we are working with the same CyaA proteins as Fukui-Miyazaki et al. We have further shown that the binding of *B. bronchiseptica* CyaA on myeloid cells is inhibited by the monoclonal antibody M1/70 against the CD11b subunit of CD11b/CD18, as has been repeatedly demonstrated for recombinant CyaA^23,24^. All these data indicate that *B. pertussis* and *B. bronchiseptica* CyaAs use the same mechanisms required for receptor binding, membrane insertion, and subsequent penetration into CD11b/CD18-positive cells.

We have previously shown that the recombinant CyaA Y940A and CyaA Y940P variants overproduced in *E. coli* exhibit very low membrane binding and membrane penetration capacity similarly as non-acylated proCyaA^49,55^. However, analysis of the acylation status of the CyaA Y940A and CyaA Y940P variants showed that the residues K860 and K983 were correctly modified by the CyaC acyltransferase as intact CyaA^12^. This excluded the possibility that the absence of fatty-acyl chains induces destabilization of the apolar segments of the hydrophobic domain and of most of the acylation region^56^. Similarly, all three other RTX hemolysins characterized here and their corresponding mutant variants were properly acylated by a combination of myristoyl and hydroxymyristoyl chains, ruling out the possibility that the reduced cytotoxic capacity of the hemolysin mutants  was due to their aberrant acylation. This is also the first report showing that *A. pleuropneumoniae* ApxIA hemolysin, like other *bona fide* hemolysins^2^, is acylated by a mixture of myristoyl and hydroxymyristoyl fatty acyl chains.

The role of aromatic residues having a specific affinity for a membrane region near the lipid carbonyl has been shown in the membrane insertion of numerous bacterial toxins^57–59^. In case of CyaA, however, the presence of the aromatic side chain at position 940 plays rather a role in the proper folding of secondary structures of the acylated segment, preventing the formation of inactive aggregates^60^, than in the direct anchoring of the toxin into the lipid bilayer. While the Y940 residue could be functionally replaced by an aromatic phenylalanine residue, lacking hydroxylation in the para position of the aromatic ring, its replacement by an aromatic tryptophan residue resulted in reduction of the cell-binding and membrane penetration capacity of the CyaA Y940W variant (Supplementary Figure S6). This could be due to the presence  larger indole ring of the tryptophan residue that might interfere with functional folding of the acylated domain of the toxin. The membrane penetration process of CyaA may further proceed by the insertion of several putative transmembrane α-helices located in the N-terminal part of the hemolysin moiety of the toxin, comprising the hydrophobic pore-forming domain and the 'AC-to-Hly' linker segment^6,7,10,12–15,61^. Consistent with our previously published results with recombinant CyaA^12^, our recent results confirm that the tyrosine-to-phenylalanine substitution of the conserved tyrosine of the acylated domain plays no role in membrane insertion and penetration of different RTX hemolysins. However, unlike for CyaA, the tyrosine-to-alanine substitution does not reduce the cytolytic capacity of RtxA and ApxIA hemolysins no more by a factor of ~ 2. A negligible, if any, reduction of the pore-forming and cytotoxic capacity of HlyA Y643A also fits well with the results showing that membrane binding of HlyC-activated CyaA_1-501_/HlyA_1-1024_ Y643A resembles the membrane binding capacity of the intact chimeric CyaA_1-501_/HlyA_1-1024_ construct. These data together with data from CD spectra show that the presence of the aromatic ring in position 643 is not essential for proper folding of the acylated segment of HlyA and subsequent insertion of the HlyA into the lipid bilayer. However, the tyrosine-to-proline substitution most likely disrupts the secondary structure(s) of the acylated segment involved in membrane insertion, as the specific hemolytic and/or pore-forming activity of HlyA Y643P, ApxIA Y639P, or RtxA Y642P was drastically reduced compared to the intact HlyA, ApxIA or RtxA. This is most likely due, at least in the case of HlyA, to the reduced membrane binding capacity of the HlyA Y643P construct, since the pore properties, namely pore conductance and pore lifetime, of the HlyA mutant variants were similar to those of intact HlyA. Comparable pore characteristics may also indicate that the membrane-interacting pore-forming domain of HlyA Y643P is properly folded. Surprisingly, the large reduction in the ability of HlyA Y643P to insert into the plasma membrane was not reflected in the calcium-dependent folding of the truncated variant of HlyA analyzed by CD spectroscopy. This may indicate that the changes in the secondary structure of the HlyA419 Y643P construct are not as dramatic as in the similar CyaA719 Y940P construct.

The vicinity of the conserved Y940 residue of the acylated segment of CyaA is different compared to other RTX hemolysins. In contrast to CyaA, where the conserved Y940 is adjacent to a hydrophilic S939, the Y638-Y639, Y642-Y643, and F641-Y642 pairs are present in the acylated segment of ApxIA, HlyA, and RtxA, respectively. The complete loss of hemolytic and cytotoxic activity of the HlyA Y642P + Y643P variant compared with the HlyA Y642P and HlyA Y643P constructs may indicate that the non-conserved Y642, together with the conserved Y643, may maximize the cytolytic and cytotoxic effects of HlyA. Our data also suggest that *bona fide* hemolysins, such as HlyA, RtxA, and ApxIA, may interact with the plasma membrane of target cells in a different manner than CyaA and that, unlike Y940 in CyaA, the aromatic rings of Y639 in ApxIA, Y643 in HlyA or Y642 in RtxA  are not involved in the folding of the acylated segment preceding the membrane insertion of the hemolysin molecule. Sequence alignments revealed a number of other conserved or non-conserved aromatic residues within the acylated segment of RTX hemolysins^62^, which may be involved in the efficient folding of the acylated segment and the correct positioning of hemolysin molecule towards the lipid bilayer, followed by the insertion of the acylated segment or of its part into the target cell membrane.

## Methods

### Antibodies

A mouse anti-RTX monoclonal antibody 9D4 was purchased from Santa Cruz Biotechnology (Dallas, Texas). The CD11b-specific monoclonal antibody M1/70 was purchased from B.D. Biosciences Pharmingen (San Jose, CA).

### Bacterial strains and growth conditions

*B. pertussis* strains were derived from strain Tohama I obtained from Institute Pasteur collection (CIP 81.32). *B. bronchiseptica* RB50 was generously provided by Branislav Vecerek (Institute of Microbiology, Prague, Czech Republic). Bacteria were grown on (BG) agar plates (Difco, USA) supplemented with 1% glycerol and 15% defibrinated sheep blood (LabMediaServis, Jaromer, Czech Republic) at 37 °C and 5% CO_2_ for 48 h (*B. bronchiseptica*) or 72 h (*B. pertussis*) to visualize hemolysis. Liquid cultures for infection experiments were obtained by growing bacteria in modified Stainer–Scholte medium (SSM) supplemented with 3 g/l of Casamino Acids and 1 g/l of heptakis (2,6-di-O-dimethyl) β-cyclodextrin to a mid-exponential phase (*B. pertussis* OD_600_ 1.0) at 37 °C.

### Construction of *B. pertussis* and *B. bronchiseptica* mutants

*B. pertussis* and *B. bronchiseptica cya*A mutants were prepared using allelic exchange method as described previously^63^. The pSS4245 vector was a kind gift of Scott Stibitz (U.S. CBER, FDA). Site directed mutations (Y940P, Y940A and Y940F) were introduced into the *cya*A gene from *B. pertussis* by PCR mutagenesis as previously described^12^. The respective mutated *cya*A gene segments from *B. pertussis* were cloned into exchange vector plasmid pSS4245 using the *Spe*I and *Sac*I sites, yielding three different recombinant plasmids (pSS4245_CyaA Y940P, pSS4245_CyaA Y940A and pSS4245_CyaA Y940F). For *B. bronchiseptica*, only the CyaA-Y940P mutant was constructed. The site directed mutation was introduced into *cya*A gene of *B. bronchiseptica* by standard PCR mutagenesis using *B. bronchiseptica*chromosome which differs from *B. pertussis* in this *cya*A gene. The amplified PCR fragments were cloned into pSS4245 vector in the *Spe*I and *Sac*I sites, yielding recombinant plasmid pSS4245_CyaA Y940P Bb. The following primers were used: 940 SpeI for. GGACTAGTGGGTGCAGACGACAGAGA, 940 SacI rev. GGGAGCTCATGCCCGTGTCGCCCAT, Bb Y940P for. CCAACACGGTCAGCCCCGCAGCGCTGGGTCGACAGGATTCC, Bb Y940P rev. CCTGTCGACCCAGCGCTGCGGGGCTGACCGTGTTGGTGC. The mutated *cya*A genes were marked by silent mutations introducing restriction sites for the *Eco47*III, *Not*I, and *Hind*III enzymes, respectively, which enabled straightforward verification of the presence of mutations by PCR analysis. All plasmid contructs were verified by DNA sequence analysis, as well as the *cya*A locus in the chromosome of the resulting *B. pertussis* and *B. bronchiseptica* mutant strains to confirm the presence of the desired mutations. Production of CyaA was analyzed by Western blotting of whole bacterial cell lysates, using an anti-RTX monoclonal antibody (9D4) specific for CyaA^64^.

### Construction of RtxA, HlyA, ApxIA, RTX719, HlyA419 and CyaA_1-501_/HlyA_1-1024_ variants

Plasmid pT7rtxC-rtxA was used for co-expression of the *rtxC* and *rtxA* genes for production of the recombinant RtxC-activated RtxA toxin equipped with a C-terminal double 6xHis purification tag^35^. To produce HlyC-activated HlyA with a C-terminal double 6xHis tag, the plasmid pT7hlyC-hlyA was used^65^. For production of the ApxIC-acylated ApxIA toxin with 6xHis tags on both the N-terminal and C-terminal ends, the pET28bapxIC-apxIA construct was used^38^. Plasmid pT7hlyC-cyaA_1-501_-hlyA_1-1024_ was used to produce HlyC-activated CyaA_1-501_/HlyA_1-1024_ hybrid molecule^66^. The expression vectors encoding the RTX719 protein was derived from pT7CT7ACT1-ΔNdeI, a bicistronic vector encoding the structural *cyaA* gene and the *cyaC* gene for the dedicated acyltransferase^67^. For construction of the pT7CT7-RTX719, the PCR fragments amplified from pT7CT7ACT1-ΔNdeI using the forward 5’-ATACATATGCATCATCATCATCATCATGAAAAGCTGGCCAACGATTAC-3’ and the reverse 5’-CCAGAGCTCGTTGTCCTGG-3’ primers. Oligonucleotide-directed PCR mutagenesis was performed to construct: (i) pT7rtxCrtxA-derived plasmids for the expression of RtxC-acylated RtxA mutant variants with single substitutions Y642A, Y642F, or Y642P; (ii) pT7hlyC-hlyA-derived plasmids for the expression of HlyC-activated HlyA mutants with single/double substitutions Y642P, Y643A, Y643F Y643P, or Y642A + Y643A and Y642P + Y643P; (iii) pET28bapxIC-apxIA-derived constructs for the production of ApxIC-acylated ApxIA variants harboring single substitutions Y639A, Y639F, or Y639P; and (iv) pT7hlyC-cyaA_1-501_-hlyA_1-1024_-derived plasmids for the expression of HlyC-activated CyaA_1-501_/HlyA_1-1024_ hybrid molecules carrying single substitutions Y643A, Y643F, or Y643P.

The pT7CT7ACT1 plasmid^67^, harboring the *cyaC* and *cyaA* genes, was used to generate a construct for the expression of the HlyC-activated HlyA fragment harboring residues 419–1024 (HlyA419) and equipped with an N-terminal hexahistidine (6xHis) purification tag. For this purpose, the *cyaC* ORF in pT7CT7ACT1 was replaced from its start to stop codon by a coding sequence of *hlyC* and similarly, the *cyaA* ORF was replaced by a *hlyA* sequence encoding the residues 419–1024 of HlyA. The *hlyC* and *hlyA* sequences were PCR-amplified from the plasmid pT7hlyC-hlyA^65^ and in addition, the *hlyA* sequence was fused in frame at the N-terminus to a sequence encoding a 6xHis purification tag, to yield the pT7CT7hlyC-N-6xHis-hlyA419-1024 plasmid. Site directed mutations (Y642P, Y643P and Y643A) were introduced into the *hlyA* fragment of pT7CT7hlyC-N-6xHis-hlyA419-1024 by site-directed PCR mutagenesis as previously described^10^.

### Production and purification of RtxA, HlyA, ApxIA, RTX719 and HlyA419 variants

The RtxA, HlyA and ApxIA toxin variants were produced in *E. coli* BL21/pMM100 cells transformed with the appropriate plasmids as described earlier^65^. 500-ml cultures were grown with shaking at 37 °C in MDO medium (yeast extract, 20 g/l; glycerol, 20 g/l; KH_2_PO_4_, 1 g/l; K_2_HPO_4_, 3 g/l; NH_4_Cl, 2 g/l; Na_2_SO_4_, 0.5 g/l; thiamine hydrochloride, 0.01 g/l) containing 150 μg/ml of ampicillin and 12.5 μg/ml of tetracycline. At OD_600_ ~ 0.8, cultures were induced with 1 mM IPTG and grown for additional 3–4 h. The cells were harvested by centrifugation, washed with 50 mM Tris–HCl (pH 8.0), disrupted by sonication at 4 °C and the homogenate was centrifuged at 20,000 × g for 30 min at 4 °C. The inclusion bodies were solubilized with 50 mM Tris–HCl (pH 8.0) containing 8 M urea and the urea extract was cleared at 20,000 × g for 30 min at 4 °C. The urea extracts were loaded on a Ni–NTA agarose column (Qiagen, Germantown, MD) equilibrated with TNU buffer (50 mM Tris–HCl (pH 8.0), 200 mM NaCl and 8 M urea). The column was washed with TNU buffer containing 20 mM imidazole and the toxin variants were eluted with TNU buffer containing 250 mM imidazole. The RTX719 variants were produced in *E. coli* XL-1 Blue and purified from the urea extract on DEAE-Sepharose column (Sigma, USA) equilibrated with a buffer containing 50 mM Tris–HCl (pH 8.0), 8 M urea and 120 mM NaCl (TUN). The column was washed with TUN buffer and CyaA719 proteins were eluted with the TU buffer supplemented with 300 mM NaCl. The HlyA-derived HlyA419 variants were produced in *E. coli* XL-1 Blue, purified from the urea extract on Ni–NTA agarose and eluted from the column with TNU buffer containing 600 mM imidazole. For CD analysis, the imidazole was removed from the samples on Amicon Ultra 10 k (Merck, Germany). The purity of the proteins was monitored by SDS–polyacrylamide gel electrophoresis and protein concentrations were determined by Bradford assay (Bio-Rad, USA).

### Preparation of urea extracts for CyaA toxin assays

For preparation of urea extract for CyaA assays, *B. pertussis* and *B. bronchiseptica* strains were grown in 20 ml SSM containing 20% L-glutamate and without addition of FeSO_4_.7H_2_O. The SSM was supplemented with 3 g/l of Casamino Acids. No cyclodextrin was added. The bacteria were grown to late exponential/early stationary phase (*B. bronchiseptica* OD_600_ 3.0–4.0, *B. pertussis* OD_600_ 1.5–2.0). Cultures were collected by centrifugation (20 min at 30 000 × g) and pellets were resuspended in 500 µl of 4 M urea and 50 mM Tris–HCl (pH 8). The mixture was incubated at 4 °C for 30 min with rotation. The urea extracts were then cleared by centrifugation (15 000 × g for 10 min) and supernatants were used for determination of AC and CyaA toxin activities.

### Animal infection experiment

Five-week-old female Balb/cByJ mice (Charles River, France) were used in this study. Mice were anesthetized by intraperitoneal (i.p.) injection of ketamine (80 mg/kg) and xylazine (8 mg/kg) and intranasally infected with the indicated colony forming units (CFUs) of mid-exponential phase *B. pertussis* CIP 81.32 or its *B. pertussis* CyaA Y940P, *B. pertussis* CyaA Y940F and *B. pertussis* CyaA Y940A derivatives delivered in 50 μl volume. To determine viable CFUs of the *B. pertussis* inoculum, aliquots of the inoculum were diluted in PBS and plated on BG agar plates. For survival experiments (LD_50_), groups of six Balb/cByJ mice were infected with serially diluted bacterial suspension and their survival was monitored over a 10-day period. The LD_50_ values were calculated by the probit analysis method of Finney, as previously reported^68^. 

### Ethics statement

The study was carried out in compliance with the ARRIVE guidelines. All animal experiments were approved by the Animal Welfare Committee of the Institute of Molecular Genetics of the Czech Academy of Sciences, v. v. i., in Prague, Czech Republic. Handling of animals was performed according to the *Guidelines for the Care and Use of Laboratory Animals*, the Act of the Czech National Assembly, Collection of Laws no. 246/1992. Permission no. 41/2019 was issued by the Animal Welfare Committee of the Institute of Molecular Genetics of the Czech Academy of Sciences in Prague.

### Assay of AC activity

Adenylate cyclase enzymatic activity was measured in the presence of 1 µM calmodulin as previously described^69^. One unit of adenylate cyclase corresponds to 1 μmol of cAMP formed in 1 min at pH 8 at 30 °C.

### Binding and cell-invasive activities on sheep RBCs

Sheep erythrocytes (LabMediaServis, Czech Republic) were washed in TNC buffer (50 mM Tris–HCl at pH 7.4, 150 mM NaCl, 2 mM CaCl_2_), adjusted to 5 × 10^8^ cells/ml, and incubated with CyaA extracts at 37 °C in TNC buffer. After 30 min, cells were washed in TNC buffer to remove unbound CyaA and divided into two aliquots. One aliquot was after extensive washing in cold 50 mM Tris–HCl at pH 7.4, 150 mM NaCl, and 5 mM EDTA (TNE buffer) directly used to determine the amount of cell-associated AC activity (Binding, membrane-bound CyaA). The other aliquot was treated with 20 µg/ml of trypsin for 15 min at 37 °C in order to inactivate the extracellular AC enzyme that did not translocate across the cellular membrane. Soybean trypsin inhibitor (40 µg/ml) was then added to the mixture to stop the reaction before the samples were washed twice in cold TNE buffer and used to determine the amount of cell-invasive AC enzyme activity^6^. The activity of intact CyaA was taken as 100%.

### Binding of CyaA to THP-1 cells

THP-1 cells (10^6^/ml) were incubated in Dulbecco's Modified Eagle Medium (D-MEM) with CyaA variants for 30 min at 4 °C, before unbound toxin was removed by washing three times with D-MEM. Cells were lysed with 0.1% Triton X-100 and membrane-associated AC enzyme activity was determined as above.

### cAMP determination

THP-1 cells were incubated at 37 °C with CyaA for 30 min in D-MEM, the reaction was stopped by addition of 0.2% Tween-20 in  50 mM HCl, samples were boiled for 15 min at 100 °C, neutralized by addition of 150 mM unbuffered imidazole and cAMP was measured by a competitive immunoassay as previously described^70^. Activity of CyaA was taken as 100%.

### Hemolytic activity on sheep erythrocytes

Hemolytic activity was measured by determining the hemoglobin release (A_541_) upon HlyA, ApxIA or RtxA incubation with 5 × 10^8^/ml washed sheep erythrocytes in TNC buffer.

### Cell viability assay

Cell viability of THP-1 cells after exposure to HlyA, was determined in D-MEM medium as the capacity of mitochondrial reductases to convert the tetrazolium salt WST-1 (4-[3-(4-iodophenyl)-2-(4-nitrophenyl)-2H-5-tetrazolio]-1,3-benzene disulfonate) to formazan, using the WST-1 assay kit (Roche) according to the manufacturer's.

### Cell lines

Human monocytes THP-1 cells (ATTC number TIB-202) were cultured at 37 °C in a humidified air/CO_2_ (19:1) atmosphere in RPMI medium supplemented with 10% heat-inactivated fetal bovine serum (FCS), penicillin (100 I.U./ml), streptomycin (100 μg/ml) and amphotericin B (250 ng/ml). Prior to assays, RPMI was replaced with D-MEM medium (1.9 mM Ca^2+^) without FCS.

### Liquid chromatography-mass spectrometry (LC–MS) analysis

Proteins were dissolved in 50 mM ammonium bicarbonate buffer (pH 8.2) to reach 4 M concentration of urea and digested with trypsin (Promega, Madison, WI, modified sequencing grade) at a trypsin:protein ratio of 1:50 for 6 h at 30 °C. The second portion of trypsin was added to a final ratio of trypsin:protein of 1:25 and the reaction was carried out for another 6 h at 30 °C. When the reaction was complete, the concentration of the resulting peptides was adjusted by 0.1% trifluoroacetic acid (TFA) to 0.1 mg/ml and 5 µl of the sample were injected into the LC–MS system. The LC separation was performed using a desalting column (ZORBAX C18 SB-300, 0.1 × 2 mm) at a flow rate of 40 µl/min (Shimadzu, Kyoto, Japan) of 0.1% FA and a separation column (ZORBAX C18 SB-300, 0.2 × 150 mm) at a flow rate of 10 µl/min (Agilent 1200, Santa Clara, CA) of water/acetonitrile (MeCN) (Merck, Darmstadt, Germany) gradient: 0–1 min 0.2% formic acid (FA), 5% MeCN; 5 min 0.2% FA, 10% MeCN; 35 min 0.2% FA, 50% MeCN; 40 min 0.2% FA, 95% MeCN; 40–45 min 0.2% FA, 95% MeCN. A capillary column was directly connected to a mass analyzer. The MS analysis was performed on a commercial solariX XR FTMS instrument equipped with a 15 T superconducting magnet and a Dual II ESI/MALDI ion source (Bruker Daltonics, Billerica, MA, USA). The mass spectra of the samples were obtained in the positive ion mode in an m/z range of 150–2000. The accumulation time was set to 0.2 s, LC acquisition was 45 min with 5 min delay and one spectrum consisted of the accumulation of four experiments. The instrument was operating in survey LC–MS mode and calibrated online using Agilent tuning mix, which results in mass accuracy below 2 ppm. After analysis, spectra were processed using the Data Analysis 4.4 software package (Bruker Daltonics) and extracted data were searched against the FASTA of single corresponding toxin molecule (ApxIA, UniProtKB: P55128; HlyA, UniProtKB: P08715; RtxA, UniProtKB: A0A1X7QMH9) using Linx software (RRID:SCR_018657). The Linx algorithm was set for fully tryptic restriction with a maximum of 3 missed cleavages and variable modification for methionine oxidation along with lysine acylation ranging from C12 to C18, including monosaturated and hydroxylated variants. The acylation status of lysine residues was determined by comparison of relative intensity ratios between acylated peptide ions and their unmodified counterparts^2^.

### CD spectroscopy

The far-UV CD spectra were recorded at 25 °C on a Chirascan-plus spectrometer (Applied Photophysics, USA) in rectangular quartz Suprasil cells of 1-mm path length (110-QS, Hellma, Germany). Protein samples were diluted in 5 mM Tris–HCl (pH 8.0) and 20 mM NaCl in the absence or presence of CaCl_2_ and measured at wavelengths from 203 to 260 nm at a scan speed of 1 nm/s. Ca^2+^-induced structural changes were monitored by stepwise titration of protein samples with increasing concentrations of CaCl_2_. The spectra of the buffers were subtracted from the protein spectra and the molar residue ellipticity (Θ) was expressed in milidegrees square centimeter per decimole [mdeg cm^2^ dmol^−1^].

### Gel filtration

Gel filtration chromatography was performed on a Superdex 200HR gel filtration column (GE Healthcare, UK) connected to AKTAprime Plus liquid chromatography system (GE Healthcare, UK). The column was equilibrated with a buffer containing 20 mM Tris–HCl (pH 8.0), 150 mM NaCl and 2 mM CaCl_2_ before the denatured proteins (400 µg) in 50 mM Tris–HCl (pH 8.0) and 8 M urea were loaded onto the column and refolded at a flow rate of 0.5 ml/min.

### Planar lipid bilayers

Measurements on planar lipid bilayers were performed in Teflon cells separated by a diaphragm with a circular hole (diameter 0.5 mm) bearing the membrane^15^. RTX proteins were prediluted in TUC buffer (50 mM Tris–HCl (pH 8.0), 8 M urea, and 2 mM CaCl_2_) and added to the grounded cis compartment with positive potential. The membrane was formed by the painting method using soybean lecithin in n-decane–butanol (9:1, vol/vol). Both compartments contained 150 mM KCl, 10 mM Tris–HCl (pH 7.4), and 2 mM CaCl_2_, the temperature was 25 °C. The membrane current was registered by Ag/AgCl electrodes (Theta) with salt bridges (applied voltage, 50 mV), amplified by LCA-200-100G and LCA-200-10G amplifiers (Femto, Berlin, Germany), and digitized by use of a LabQuest Mini A/D convertor (Vernier, Beaverton, OR). For lifetime determination, approximately 400 of individual pore openings were recorded and the dwell times were determined using QuB software with 100 Hz low-pass filter. The kernel density estimation was fitted with exponential function using Gnuplot software. The relevant model was selected by the χ2 value.

### Statistical analysis

Results were expressed as the arithmetic mean ± standard deviation (SD) of the mean. One- or two-way ANOVA was used to calculate statistical significance (GraphPad Prism 9.1.1; GraphPad Software, La Jolla, CA). Significant differences from intact toxin are indicated by asterisks (*p < 0.05; **p < 0.01; ***p < 0.001; ****p < 0.0001).

## Supplementary Information

Supplementary Information.

## Data Availability

All data generated or analysed during this study are included in this published article (and its Supplementary Information file).
