# Supplementary material for: Different roles of conserved tyrosine residues of the acylated domains in folding and activity of RTX toxins
Source: Sci Rep. 2021 Oct 6;11:19814. doi: 10.1038/s41598-021-99112-3 (PMC8494930; doi:10.1038/s41598-021-99112-3)
Supplement: Supplementary file 1 — Supplementary Information. [file 41598_2021_99112_MOESM1_ESM.pdf]

Supplementary Information

Different roles of conserved tyrosine residues of the acylated domains in folding and activity of RTX toxins

Anna Lepesheva, Adriana Osickova, Jana Holubova, David Jurnecka, Sarka Knoblochova, Carlos Espinosa-Vinals, Ladislav Bumba, Karolina Skopova, Radovan Fiser, Radim Osicka, Peter Sebo and Jiri Masin

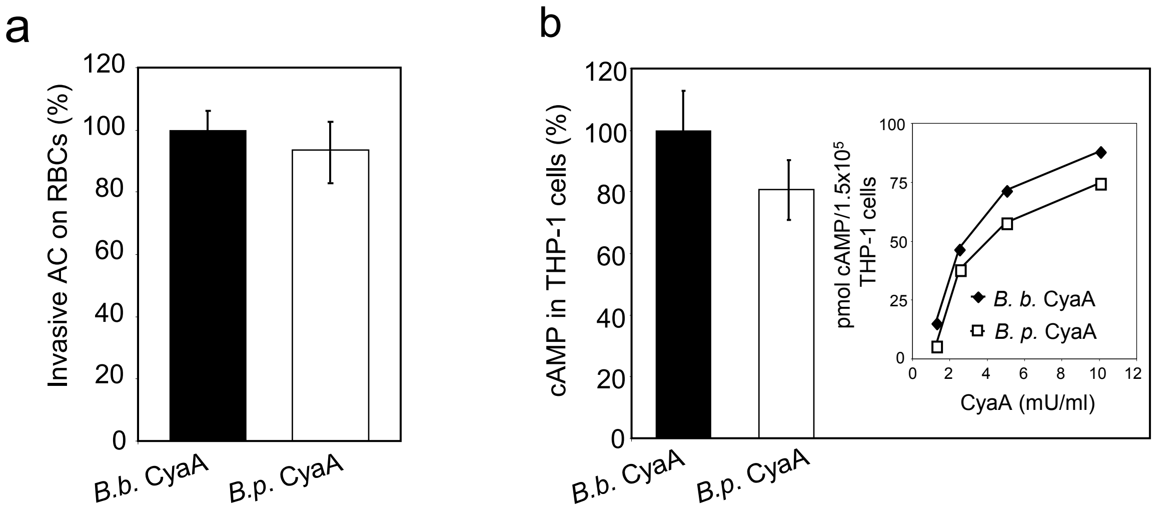

Supplementary Figure S1: Both *B.p.* and *B.b.* CyaAs show a similar capacity to deliver the AC domain across the plasma membrane. (a) For analysis of the cell-invasive activity of *B.b.* and *B.p.* CyaA, sheep erythrocytes (5×10^8/ml) were incubated at 37 °C with extracts (10 mU/ml). After 30 min, aliquots were taken to determine the AC activity internalized into erythrocytes and protected against digestion by externally added trypsin (invasive AC). Activities are expressed as percentages of intact *B.b.* CyaA activity and represent mean ± SD of three independent determinations performed in duplicate. **(b)** Translocation of the AC domain was quantified by determining the intracellular cAMP concentration measured in THP-1 cells (1.5×10^5) after incubation with *B.p.* and *B.b.* extracts (10, 5, 2.5 and 1.25 mU/ml CyaA) for 30 min at 37 °C (right panel). Activities are expressed as percentages of intact *B.b.* CyaA activity and represent mean ± SD of three independent determinations performed in duplicate with two independent preparations of extracts.

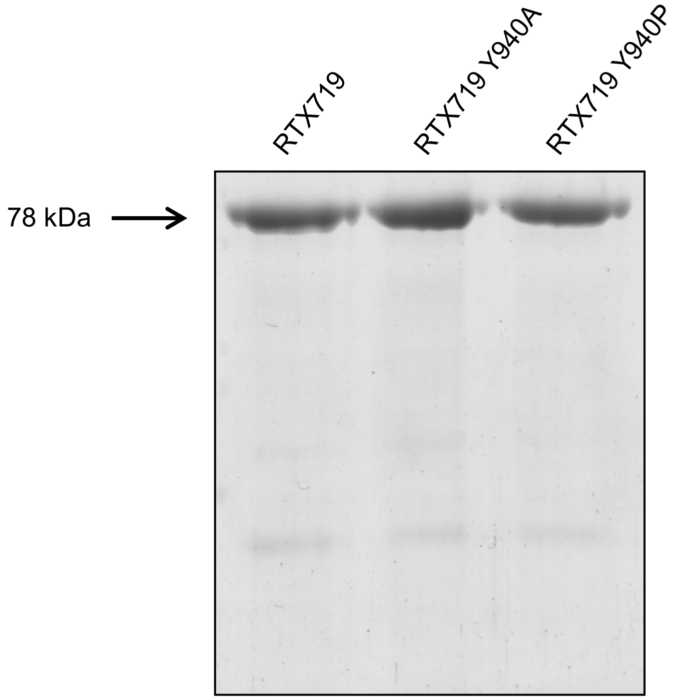

Supplementary Figure S2: SDS-PAGE analysis of the purified CyaA-derived RTX719 variants. The RTX719 variants were produced in *E. coli* XL-1 Blue and purified from the urea extract on DEAE-Sepharose column as described in Methods. The purified proteins were separated by SDS-PAGE (7.5%) and visualized by Coomassie blue staining.

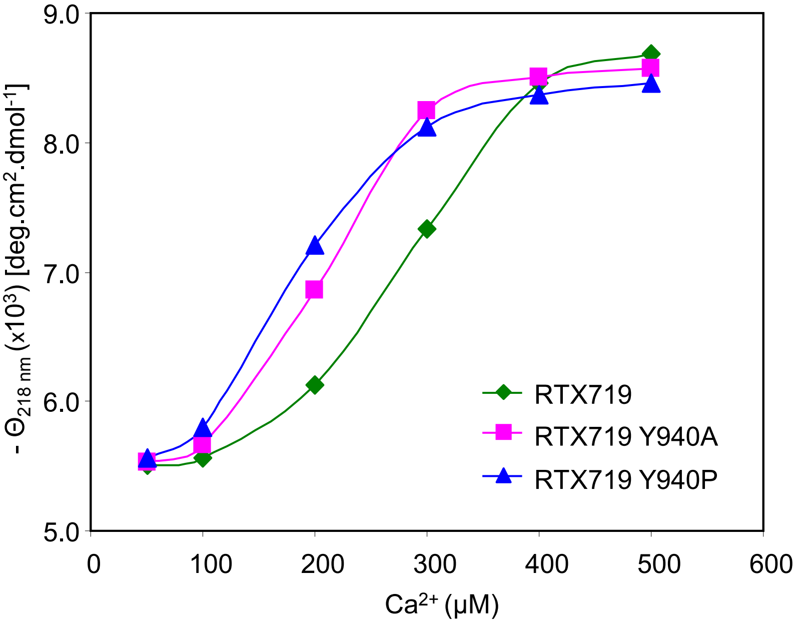

Supplementary Figure S3. The Ca^{2+}-induced folding of RTX719, RTX719 Y940A and RTX719 Y940P differs. The proteins (200 μg/ml) were titrated with $CaCl_2$, and molar ellipticity was followed at 218 nm ($\Theta_{218\ nm}$) as a function of Ca^{2+} concentration.

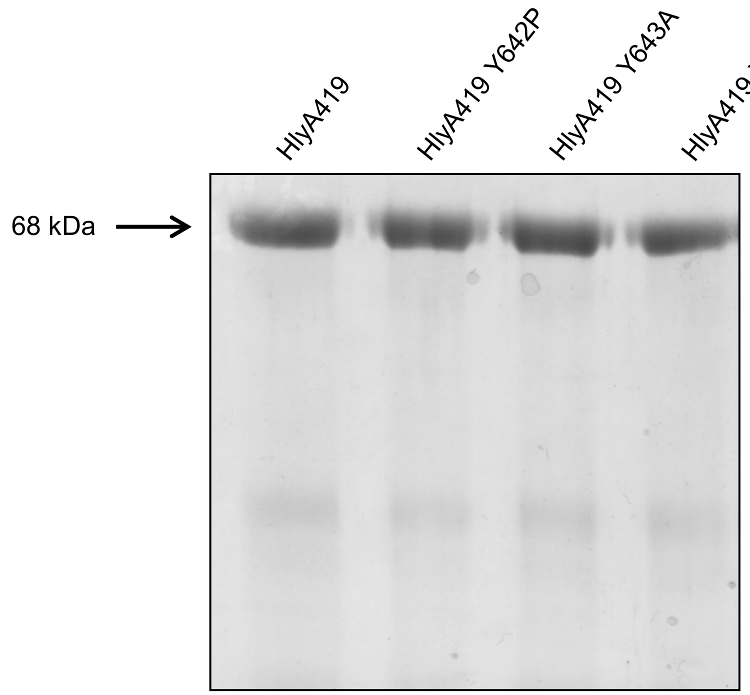

Supplementary Figure S4: SDS-PAGE analysis of the purified HlyA419 variants. The HlyA419 variants were produced in *E. coli* XL-1 Blue and purified from the urea extract on Ni-NTA agarose column as described in Methods. The purified proteins were separated by SDS-PAGE (7.5%) and visualized by Coomassie blue staining.

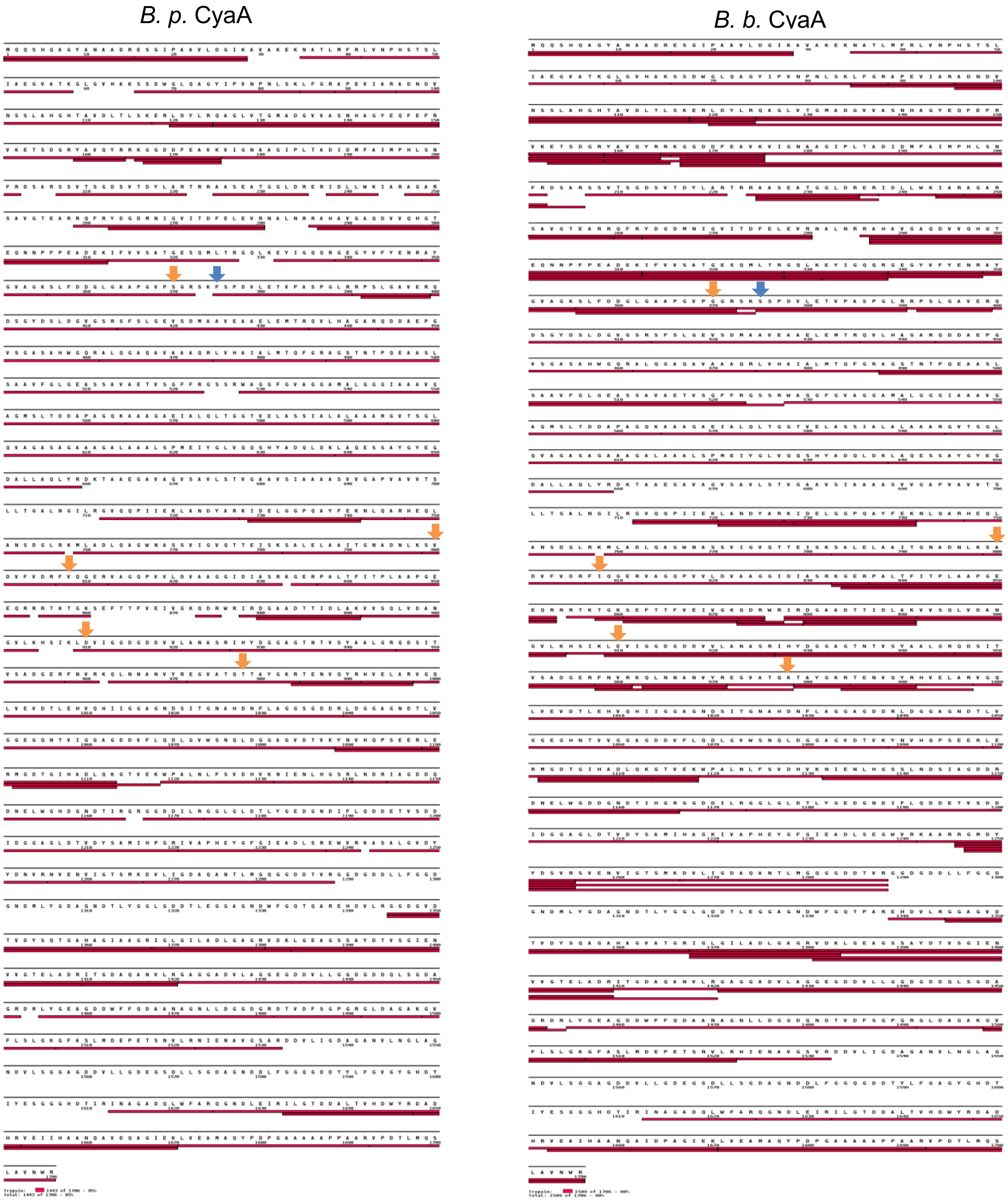

Supplementary Figure S5: Peptide coverage map of *B.p.* and *B.b.* CyaAs. Purified CyaA proteins from *B. p.* CIP 81.32 or *B. b.* RB 50 were applied to a 30-kDa-cutoff membrane filter (Millipore, Sigma, USA), washed twice with AB buffer (50 mM ammonium bicarbonate, pH 8.3) and digested by spectrometry-grade trypsin (Promega, USA) in AB buffer at a protein/enzyme ratio of 35:1 for 16 hours at 37 °C. Resulting peptides were desalted using C18 extraction disks (Empore, USA) and analyzed by liquid chromatography coupled to ultrahigh-resolution Fourier transform ion cyclotron resonance mass spectrometer (LC FT-ICR MS). The experimental data were searched against the FASTA of respective toxin molecules (*B. p.* CyaA: UniprotKB code P0DKX7; *B. b.* CyaA: UniprotKB code Q57506) and matching peptides (red rectangles) were visualized over corresponding sequence using MSTools (https://doi.org/10.1016/j.ijms.2010.07.030). Position of a phenylalanine 375 in the sequence of *B. p.* CyaA or a serine 375 in *B. b.* CyaA is highlighted by blue arrow. Position of S370, V800, V808, D910, T978 in *B.p.* CyaA and G370, A800, I808, E910, K978 in *B.b.* CyaA is highlighted by orange arrow.

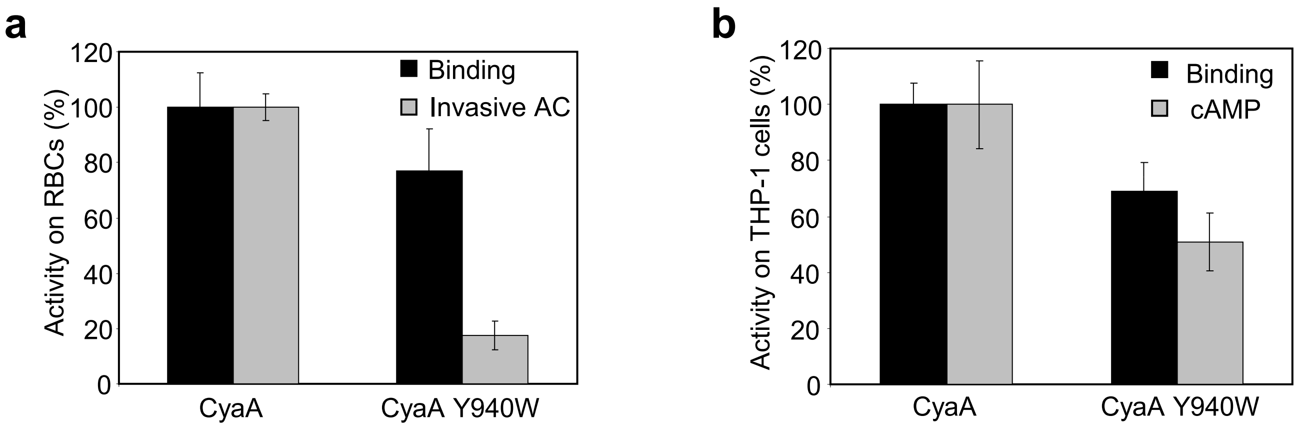

Supplementary Figure S6. Y940W substitution impairs membrane insertion and AC domain translocation of CyaA. CyaA proteins were expressed in *E. coli*. XL-1 and 8M urea extracts were prepared. **(a)** Sheep erythrocytes (5×10^8/ml in Tris 50 mM, NaCl 150 mM, $CaCl_2$ 2 mM, pH 7.4) were incubated at 37°C with urea extracts (final concentration of CyaA 0.4 U/ml). After 30 min, aliquots were taken for determination of the cell-associated AC activity (binding) and of the AC activity internalized into erythrocytes and protected against digestion by externally added trypsin (invasive AC). Activities are expressed as percentages of intact CyaA activity and represent mean ± SD, n=3. **(b)** Binding of CyaA or CyaA Y940W to THP-1 cells (10^6) was determined as the amount of cell-associated AC enzyme activity upon incubation of cells with 40 mU/ml of the protein for 30 min at 4°C. AC domain translocation was assessed by determining the intracellular concentration of cAMP generated in THP-1 cells (1.5×10^5) following incubation with diluted bacterial lysates with three different toxin concentrations from within the linear range of the dose-response curve (12.5, 6 and 3 mU/ml). Activities are expressed as percentages of intact CyaA activity and represent mean ± SD from three independent determinations performed in duplicate.

Supplementary Table S1. Acylation status of RtxA, HlyA and ApxIA variants.

protein[a]			
	modification	Lys558[b]	Lys689[b]
RtxA	non-modified	99.6	0
	C14:0	0.2	69.4
	C14:0-OH	0.2	20.2
RtxA-Y642A	non-modified	99.7	0
	C14:0	0.2	75.4
	C14:0-OH	0.1	16.9
RtxA-Y642F	non-modified	99.2	0
	C14:0	0.6	75.2
	C14:0-OH	0.2	15.5
RtxA-Y642P	non-modified	99.8	0
	C14:0	0.1	77.4
	C14:0-OH	0.1	16.0
	modification	Lys564[b]	Lys690[b]
HlyA	non-modified	4.2	12.8
	C14:0	46.2	58.0
	C14:0-OH	35.4	28.2
HlyA-Y643A	non-modified	18.8	5.5
	C14:0	32.1	54.4
	C14:0-OH	41.7	33.3
HlyA-Y643F	non-modified	18.3	8.5
	C14:0	26.5	49.8
	C14:0-OH	47.0	34.8
HlyA-Y643P	non-modified	22.4	8.8
	C14:0	35.2	54.7
	C14:0-OH	36.2	31.5
HlyA-Y642P+Y643P	non-modified	5.7	0
	C14:0	22.0	40.6
	C14:0-OH	62.2	44.3
	modification	Lys570[b]	Lys696[b]
ApxIA	non-modified	0	0
	C14:0	49.3	49.6
	C14:0-OH	40.7	34.5
ApxIA-Y639A	non-modified	0	0
	C14:0	42.9	47.2
	C14:0-OH	47.3	37.6
ApxIA-Y639F	non-modified	0	0
	C14:0	39.0	46.7
	C14:0-OH	50.2	38.2
ApxIA-Y639P	non-modified	0	0
	C14:0	31.7	44.2
	C14:0-OH	53.4	39.1

[a]Proteins were produced in the *E. coli* strain BL21/pMM100 and purified close to homogeneity as described in Methods.

[b]Percentage distribution of fatty acid modification of the ε-amino groups of the Lys570 and Lys696 residues of the ApxIA-derived constructs, the Lys564 and Lys690 residues of the HlyA-derived constructs and the Lys558 and Lys689 of the RtxA-derived constructs. The remaining Lys residues to 100% are acylated by C12:0, C12:0-OH, C14:1, C14:1-OH, C16:0 and C16:1.
